# Sintilimab plus autologous NK cells as second-line treatment for advanced non-small-cell lung cancer previous treated with platinum-containing chemotherapy

**DOI:** 10.3389/fimmu.2022.1074906

**Published:** 2022-12-08

**Authors:** Lin Jia, Naifei Chen, Xiao Chen, Chao Niu, Ziling Liu, Kewei Ma, Nanya Wang, Lei Yang, Yuguang Zhao, Wei Song, Jin Lu, Chen Chen, Xiaofeng Cong, Xu Wang, Yinghui Xu, Guozhen Cui, Zengguang Liu, Rongrong Chen, Wei Li, Jiuwei Cui

**Affiliations:** ^1^ Cancer Center, The First Hospital of Jilin University, Changchun, China; ^2^ Department of Medical Center, GenePlus-Beijing, Beijing, China

**Keywords:** non-small cell lung cancer, PD1 antibody, NK cells, immunotherapy, immune cell therapy

## Abstract

This pilot study (NCT03958097; https://www.clinicaltrials.gov/ct2/show/NCT03958097) was aimed to evaluate the efficacy and safety of PD-1 antibody combined autologous NK cells in the treatment of patients with stage IIIB/IIIC or IV non-small-cell lung cancer (NSCLC) who failed the first-line platinum-based chemotherapy. All patients received both sintilimab 200mg and 3×10^9^ NK cells every 3 weeks. 20 patients were enrolled, median follow up time was 22.6 months. The median PFS was 11.6 months, ORR was 45%. Median OS was 17.7 months, 6-month OS rate and 12-month OS rate was 95.0% and 80.0%. Unexpected adverse events were not observed. 2 patients reported grade 3 adverse events (hypertriglyceridemia, neutropenia and increased creatine kinase). The autologous NK cells did not add extra adverse events to the ICI treatment. Autologous NK plus sintilimab showed promising antitumor activity and an acceptable safety profile in advanced driven-mutation negative NSCLC who failed on the first line treatment.

## Introduction

Lung cancer remains the leading cause of cancer-related mortality in both men and women. Non-small cell lung cancer (NSCLC) is the most common variety accounting for 84% of the cases. Most patients are diagnosed at an advanced stage of the disease, for which the 5-year survival rate is only 6% (1). Immune checkpoint inhibitors (ICI) have fundamentally changed the therapeutic landscape for NSCLC, but the overall response rate (ORR) is only between 15-20%, most patients do not benefit from this treatment. Although programmed death 1 (PD-1)/Programmed death ligand 1 (PD-L1) blockade shows remarkable therapeutic effects and prolongs patient survival in the clinic, the response rate of unselected NSCLC patients is approximately 20% (2). Combination therapy has emerged as an effective way to broaden the benefits of PD-1/PD-L1 immunotherapy and overcome or delay the resistance.

Natural killer (NK) cells are effector cells of the innate immune system and belong to the family of innate lymphoid cells (ILCs). NK cells are professional killer cells that recognize and rapidly destroy cells that are dangerous to the cancer immune surveillance, and they are important in the control of cancer metastasis. NK cells can directly kill tumor cells, secrete various cytokines to initiate antitumor responses, and recruit other immune cells into the antitumor response. PD-1 was found expressed in NK cells and often induced or upregulated on tumor-infiltrating NK cells, affecting the antitumor function of NK cells. Anti-PD-1 therapy could improve their responsiveness and tumor control even in the absence of T cell cells, indicating that, in addition to T cells, other immune cells, such as NK cells, could also mediate the effect of PD-1/PD-L1 blockade immunotherapy (3, 4). Recent studies have shown that NK cells can enhance the efficacy of the anti-PD-1 antibody. NK cells also participate in the clinical benefit of PD-1/PD-L1 antibody therapy, and NK cells also have greater off-the-shelf utility and are safer than other cell-therapies (e.g. CAR-T), as they cause fewer immune-related adverse events, this study was aimed to evaluate the efficacy and safety of autologous NK cells combined with PD-1 antibody for advanced driven mutation negative NSCLC as a second-line therapy.

## Results

### Patient and treatment exposure

From May 2019 to Oct 2020, 20 patients were enrolled (**Table 1**). As of data cut-off date Nov 1, 2021, the median follow-up time was 22.6 months (range from 2.7 months to 25.9 months). All patients received at least 2 cycles of sintilimab and NK cells. As of analysis date, 12 patients had disease progression, 8 patients were still in the study, 1 patient discontinued sintilimab due to toxicity. Median treatment cycle was 11 (range from 2 to 36).

**Table 1:** Baseline characteristics and prior therapy of all treated patients with NSCLC.

Characteristic	All Treated Patients (N=20)		
	No.	%	
Age, years			
Median (range)	62(49-71)		
Sex			
Male	17	85	
Female	3	15	
Smoking status			
Never	10	50	
Smoker	10	50	
ECOG performance status			
0	1	5	
1	19	95	
Tumor cell histology			
Adenocarcinoma	8	40	
Squamous	12	60	
Previous chemotherapy regimens			
Gemcitabine + platinum	12	60	
Pemetrexed + platinum	7	35	
Paclitaxel + platinum	1	5	

### Efficacy assessments

#### Progression free survival (PFS)

**Figure 1:**
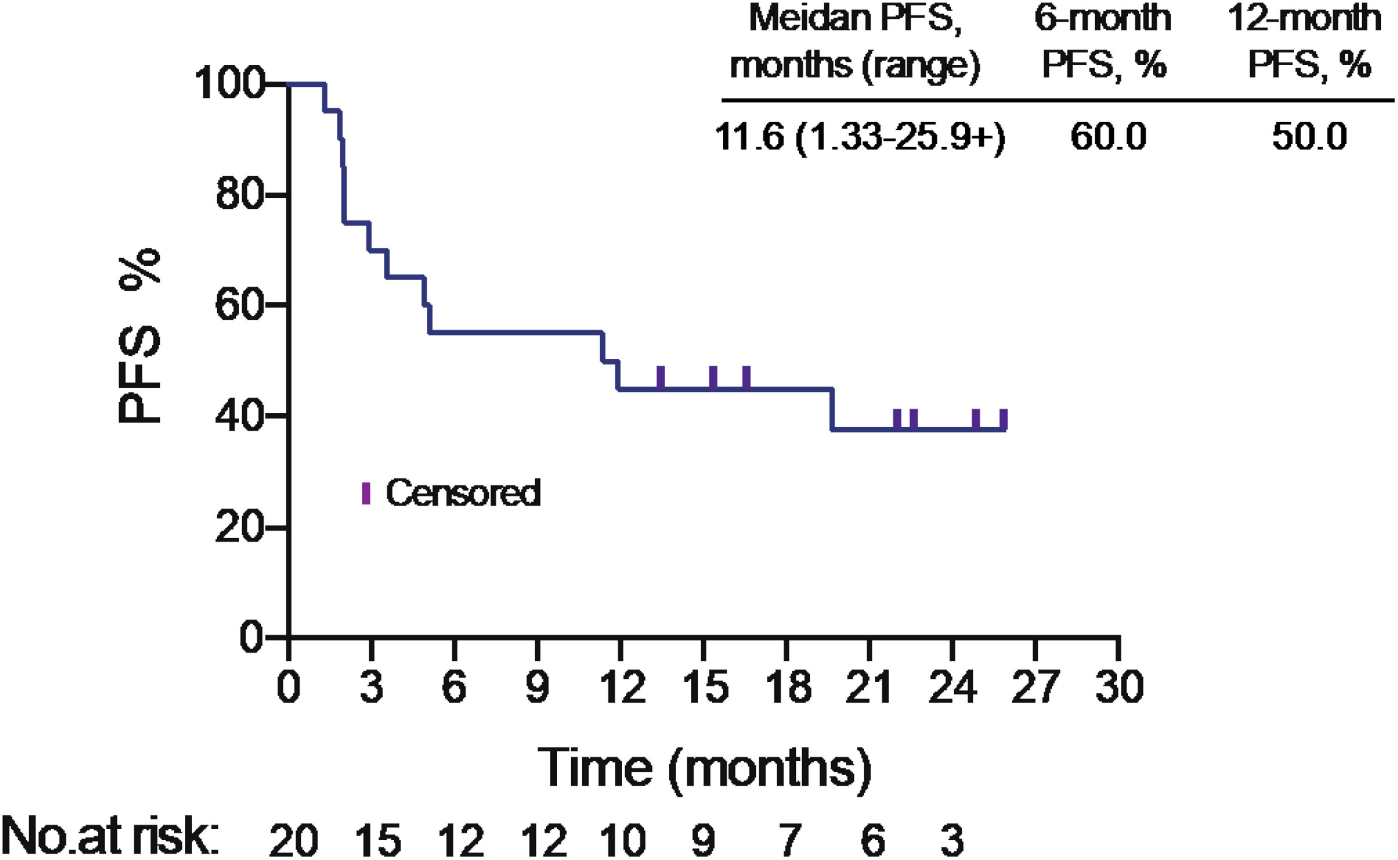
Progression-free survival in intention to treat population.

The median PFS was 11.6 months in intention to treat (ITT) population (**Figure 1**). PFS was assessed in different subgroups, such as age, smoking status, histology, tumor mutational burden (TMB) level. We found PFS was superior in squamous carcinoma patients than adenocarcinoma patients (p<0.05) (**Table S1**). Median PFS of adenocarcinoma patients was only 2.02 months (95% CI: 1.97-NA), while the PFS of squamous carcinoma patients was 19.67 months (95% CI: 11.37-NA). But no significant difference found in other subgroups.

#### Objective response rate (ORR)

**Figure 2:**
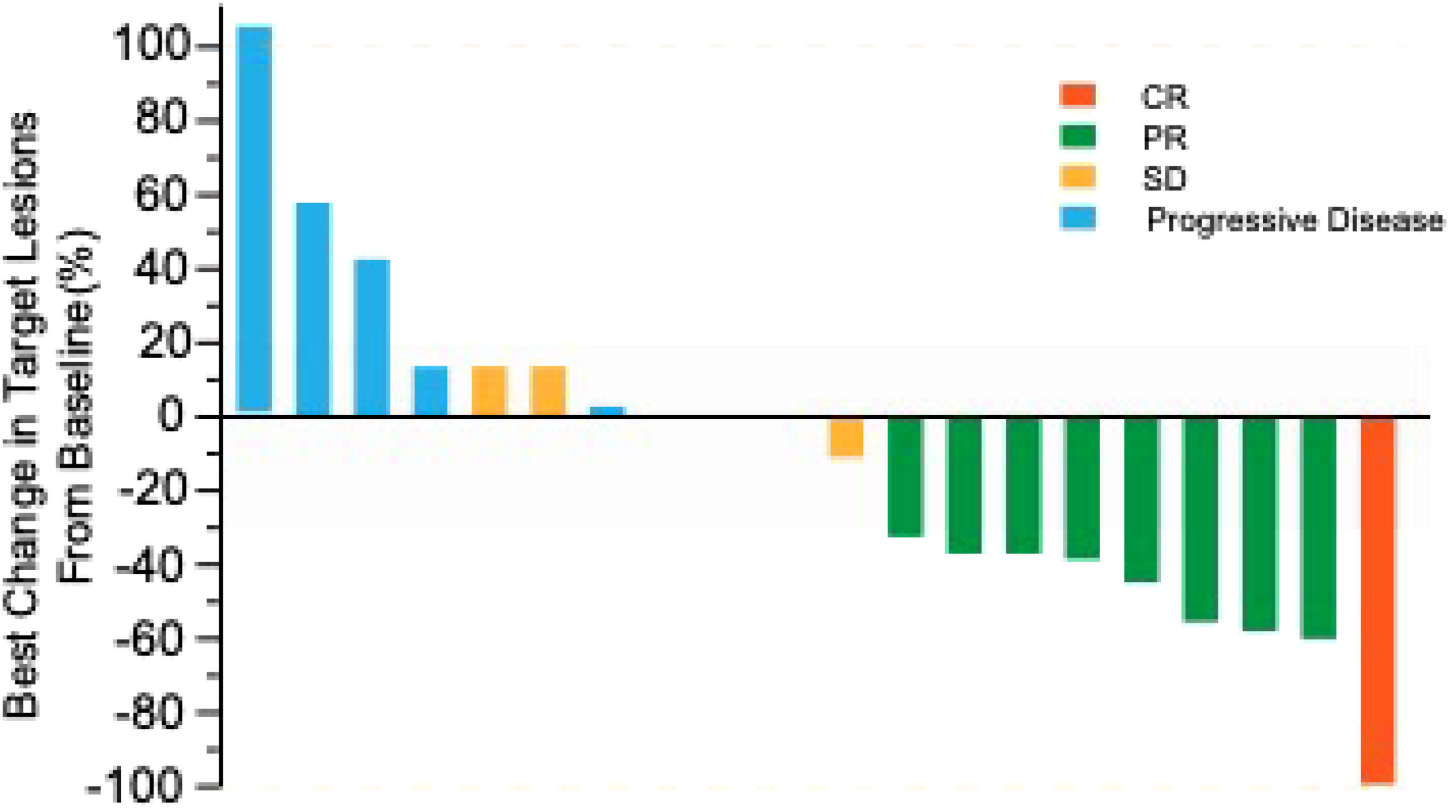
Best percentage change from baseline in target-lesion size.

**Figure 3:**
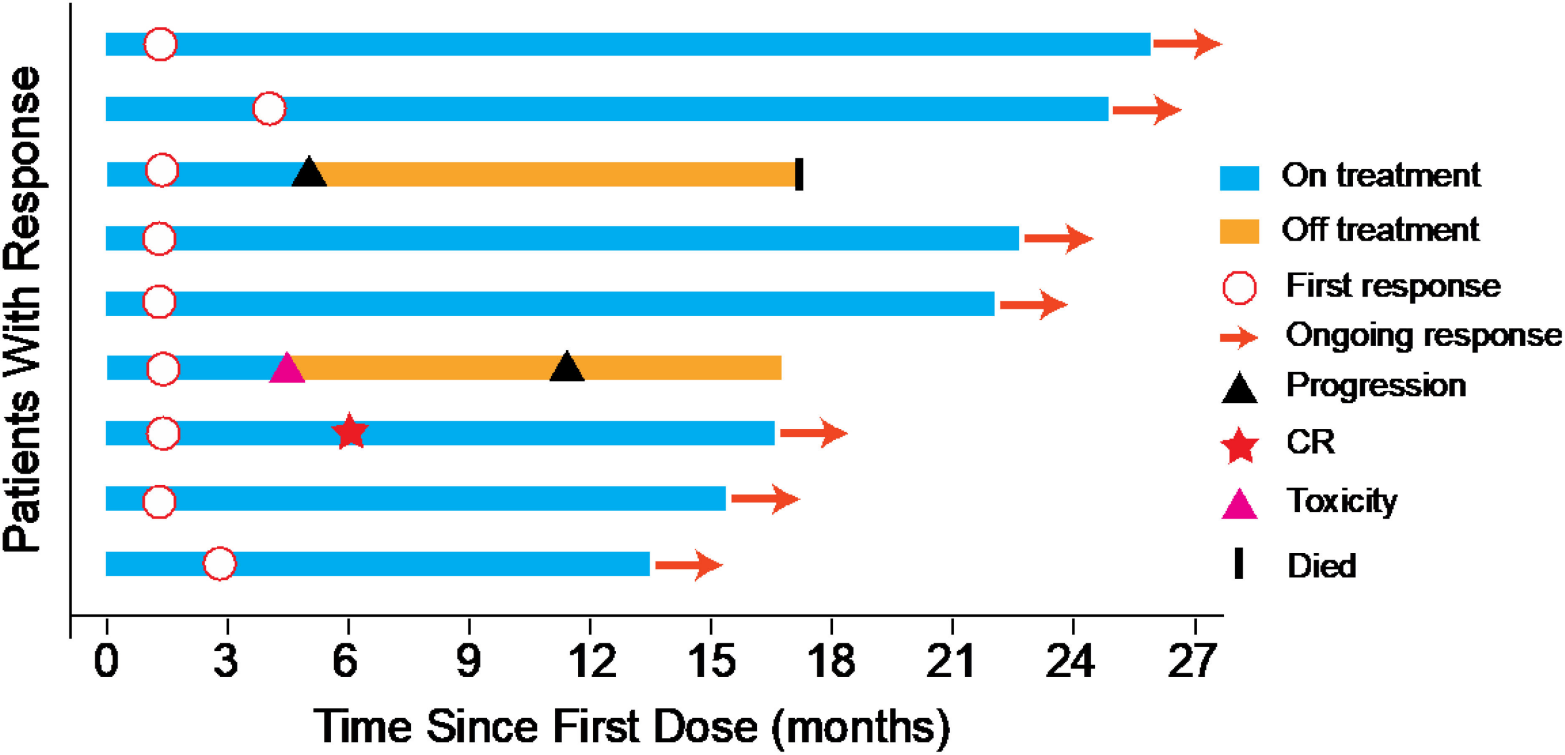
Duration of response for patients with PR and CR.

ORR was 45% evaluated by Response Evaluation Criteria in Solid Tumours (RECIST) v1.1 (**Table 2**), including 5% complete response (CR) and 40% partial response (PR). 50% of the patients had a reduction of target lesion size from baseline (**Figure 2**). In the protocol-specified response-assessable population, confirmed PR occurred in 9 of the patients. Among patients with ongoing responses (77.8%; 7/9), response durations ranged from 3.5 to 24.5+ months (**Figure 3**). Median response time was 1.4 months (range from 1.3 months to 4.0 months).

**Table 2:** Summary of response for patients with an intention-to-treat analysis for PFS and OS.

Response/Survival	All Patients (N = 20)	
	No	%
Confirmed ORR	9	45
Confirmed DCR	14	70
Ongoing responders	7	35
BOR		
Confirmed CR	1	5
Confirmed PR	8	40
SD	5	25
Progressive disease	6	30
PFS, median (range), months	11.6 (1.33 to 25.9+)	
OS, median (range), months	17.7 (2.3 to 25.9+)	

#### Overall survival (OS)

The median OS was 17.7 months (range from 2.3 months to 25.9 + months) (**Figure S1**). 6-month OS was 95.0%, 12-month OS was 80.0%.

### Safety evaluation

Adverse events (AEs) of any grade were reported in 95% (19/20) of patients, grade 3 AEs occurred in 10% (2/20) of patients, no grade 4 AEs occurred. The most common adverse events were hypoalbuminemia (45%), hypothyroidism (25%), anemia (20%), hyperglycemia (20%), and hyponatremia (20%) (**Table S2**). Most of adverse events were grade 1-2. 5.4% of adverse events were grade 3 (hypertriglyceridemia, neutrophil count decreased, increased creatine kinase). The patient with an adverse event of grade 3 hypertriglyceridemia had grade 2 hypertriglyceridemia at baseline. 2 patients suspended treatment due to grade 2 increased creatine kinase, 1 patient resumed NK cells after the recovery. The other patient discontinued treatment as creatine kinase increased to grade 3.

### Predictive biomarkers

#### TMB

Based on previous study, we determined 9 mut/Mb as cutoff level (5). We found baseline TMB levels had no significant difference between progressive disease (PD) group (6 patients) and disease control (DC) group (14 patients) (Figure S2). In our study, 71.4% (10/14) patients of DC group were TMB low.

#### Gene mutation

We compared gene mutation enrichment in PD and DC groups. For the limited sample number, we found no significantly enriched gene mutations between these groups. However, in PD group, most patients with primary resistance to NK and PD-1 therapy had relative negative predictive gene mutations: SKT11&KEAP1 mutation, ERBB2 amplification, PTEN deletion, EZR-ROS1 fusion and TSC1 double alleles nonsense mutation.

#### PD-1 and PD-L1 level on NK cells

**Figure 4:**
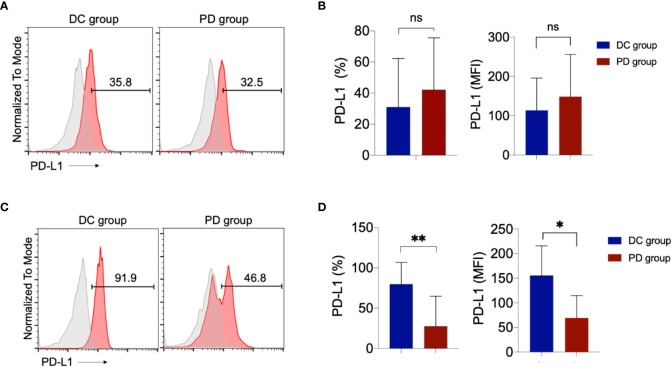
PD-L1 expression in NK cells. **(A)** Representative flow cytometry analysis of PD-L1 expression baseline. Histogram plots show the isotype control staining profile (gray line) versus the specific antibody staining profile (red line). **(B)** PD-L1+ NK cell percentages and mean fluorescence intensity (MFI) indicate the PD-L1 expression in NK cells after treatment. **(C)** Representative flow cytometry analysis of PD-L1 expression after treatment. **(D)** PD-L1+ NK cell percentages and MFI indicate the PD-L1 expression in NK cells after treatment. ns, no significance.**p<0.01.*p<0.05. .

We tested PD-1 and PD-L1 levels of NK cells. By comparing PD group and DC group, we found PD-L1 levels on NK cells at baseline had no significant difference (**Figures 4A, B**). But after treatment, PD-L1 level of NK cells in DC group were significantly higher than PD group (**Figures 4C, D**). PD-1 level of NK cells at baseline and after treatment had no significant difference between PD group and DC group (**Figure S3**).

#### Molecular Tumor Burden Index (mTBI)

We analyzed mTBI dynamically. In most patients, mTBI well matched with CT radiographic evaluation (**Figures S4–6**). As tumor size reduced, mTBI decreased (**Figure S5**). mTBI also can predict disease progression earlier than imaging change (**Figure S6**).

## Discussion

This pilot study enrolled 20 locally advanced or metastatic NSCLC patients pretreated with 1 line of platinum-based chemotherapy. In this trial, all patients received sintilimab combined with autologous NK cells, the ORR was 45%, with 1 patient (5%) achieved complete response and 8 patients (40%) achieved partial response. The median progression-free survival (PFS) was 11.6 months, and the median OS was 17.7 months.

Patients with advanced NSCLC who have failed front-line therapy continue to have poor prognosis and limited treatment options. The standard of second-line treatment for these patients has historically been platinum-based chemotherapy, immunotherapy with or without chemotherapy showed the promising results as the second-line treatment modalities. Several studies assessed the mono-immunotherapy in patients with previously treated NSCLC, the ORR ranged from 17% to 21.9%, and the PFS ranged from 3 to 4 months (6, 7). Considering the low ORR of ICIs monotherapy, current efforts have focused on exploring new potential combinatorial strategies with synergistic antitumor activity. Previous studies have demonstrated that ICIs combined with chemotherapy and/or anti-angiogenesis therapy might be a better choice. However, A prior retrospective study has evaluated the efficacy of combined therapy of PD-1 inhibitor with chemotherapy and/or bevacizumab, the ORR was only 31.8% and the median PFS was 7.5 months (8). Another study has shown that the combination of chemotherapy and PD-1 inhibitors as second-line treatment for advanced NSCLC did not improve clinical outcomes (9). Most of these studies have not yielded the desired results and the new combination strategies are needed.

NK cells have a crucial role in immunosurveillance against tumor formation. The function of NK cells are tightly regulated by a balance between activating and inhibitory signals (10). Recent studies have found the PD1 could express on the surface of NK cells. The PD1+ NK cells displayed decreased anti-tumor activity. This impairment could be partially reversed using ICIs. Previous study has demonstrated that NK cells could control the levels of stimulatory dendritic cells, which is capable of restimulating T cells in the tumor microenvironment and promote the effectiveness of ICIs immunotherapy (11). Another study has shown that PD-L1 blockade could enhance the anti-tumor function of NK cells in ICI treatment. Based on these studies, we hypothesized that PD-1 blockade could enhance the anti-tumor activity of NK cells. NK-cell-based immunotherapies have been used widely in clinical trials and have shown great promise for different hematological malignancies. However, for patients with solid tumors, the outcomes of adoptive NK cell infusions have been disappointing. Recently, Lin et al. have investigated the safety and efficacy of Pembrolizumab plus allogeneic NK cell therapy for patients with advanced NSCLC (12). The patients with PD-L1 tumor proportion score (TPS) of 1% or higher were enrolled in this study, and the allogeneic NK Cells were collected from the healthy donors who were selected based on genotyping mismatch between the KIR of allogenic donors and the HLA class I of patients. The ORR for the combination therapy was 36.4% and the median PFS was 6.5 months. In our trial, we did not select the tumor PD-L1 expression status and used the autologous NK cells from the patients, the ORR was 45% and the median PFS was 11.6 months. Our study demonstrated that the sintilimab combined with the autologous NK cells could yield an improved survival benefit for advanced NSCLC patients as the second-line therapy. Compared with the allogeneic NK Cells, the autologous NK cells without selection are easier to manipulate.

It has been reported that PD-L1 is not only expressed on tumor cells but also on immune cells, including T cells, NK cells. Previous study have shown that anti–PD-L1 antibody could activate the PD-L1+ NK cells to control growth of PD-L1 negative tumors *in vivo*, PD-L1 + NK cells could express a significant increase of granzyme B, CD107a and interferon γ after the addition of atezolizumab (13). In addition to T cells, NK cells could mediate the effect of PD-1 blockade immunotherapy. In this study, we show that PD-L1 level, not PD1 level, on NK cells in DC group were significantly higher than the PD group. The PD-L1 positive NK cells might be associated with enhanced NK-cell function and improved patients’ survival.

In our subgroup analysis, we found that the median PFS of squamous carcinoma patients was 19.67 months, but the median PFS of adenocarcinoma patients was only 2.02 months. In the ORIENT-3 study which was conducted to investigate sintilimab as second-line treatment for advanced and metastatic squamous NSCLC, the median PFS was only 4.3 months, and the overall survival was 11.79 months in the sintilimab group. The ORIENT-12 study has demonstrated that the superiority of sintilimab plus gemcitabine and platinum (GP) as first-line treatment for locally advanced or metastatic squamous non-small-cell lung cancer, the median PFS was 5.1 months, compared with 4.9 months in the GP group (14). This combination treatment as first-line treatment was significantly inferior to our strategy as second-line therapy. We could explore the possibility of our combination strategy as first line therapy for squamous carcinoma.

TMB has emerged as a potential predictive biomarker of response to immunotherapy, high TMB is associated with improved PFS in patients with NSCLC, but the mechanism between TMB and benefit from immunotherapy was not fully understood (15). In our study, 71.4% patients of DC group were TMB low. The association between NK cell therapy and TMB need be further investigated. We also found that the patients with early progression had some mutations, for example the SKT11&KEAP1 mutation, ERBB2 amplification, which previously reported as negative predictor of immunotherapy.

Circulating tumor DNA (ctDNA) is a potential biomarker of prognosis and therapeutic response, the mTBI in ctDNA could serve as a therapeutic response and prognostic biomarker in cancer treatment (16). In our study, we found that mTBI in ctDNA detected in liquid biopsies well matched with effect evaluation and could predict disease progression earlier than imaging changes. mTBI can be a potential biomarker of therapeutic response in patients treated with NK cells plus ICIs.

The most common adverse events were hypoalbuminemia, hypothyroidism, anemia, hyperglycemia, and hyponatremia, and most of these adverse events were grade 1 to 2, only 5.4% of adverse events were grade 3, no grade 4 or 5 adverse event was reported. The patient with an adverse event of grade 3 hypertriglyceridemia had grade 2 increase in triglyceride at baseline. After dietary control, the level of hypertriglyceridemia resumed to grade 2 in 3 weeks. Two patients suspended treatment due to grade 2 increased creatine kinase. 1 patient resumed therapy because creatine kinase decreased to normal level 3 weeks after treatment suspension. Patient’s creatine kinase increased to grade 2 again after received another 2 doses of sintilimab and NK cells. The patient discontinued sintilimab, but sustained NK cells, the creatine kinase decreased to normal level in 3 weeks. By the analysis date, the patient who was still in the study, the creatine kinase level of the patient remained normal. In the other case, however, creatine kinase increased to grade 3 in 3 weeks and treatment was discontinued. Three months after treatment termination, the patient’s creatine kinase level returned to normal. All the reported events were also seen in the mono-immunotherapy treatment. The addition of autologous NK cells to ICIs was well tolerated and did not cause extra adverse events compared with other combination strategies.

A major limitation of this study was the lack of the control group and the small number of patients. Another limitation was that not all the enrolled patients had the biopsy samples. Although immunotherapy has been recommended as the first-line treatment for NSCLC, there are still some patients do not receive the immunotherapy at the first-line treatment for different reasons. The encouraging results of adding NK cells to the immunotherapy suggest a possible way for the future research in the first-line treatment. In conclusion, autologous NK plus sintilimab showed promising antitumor activity and an acceptable safety profile in advanced driven-mutation negative NSCLC who failed on the chemotherapy based first line treatment.

## Methods

### Study design

**Figure 5:**
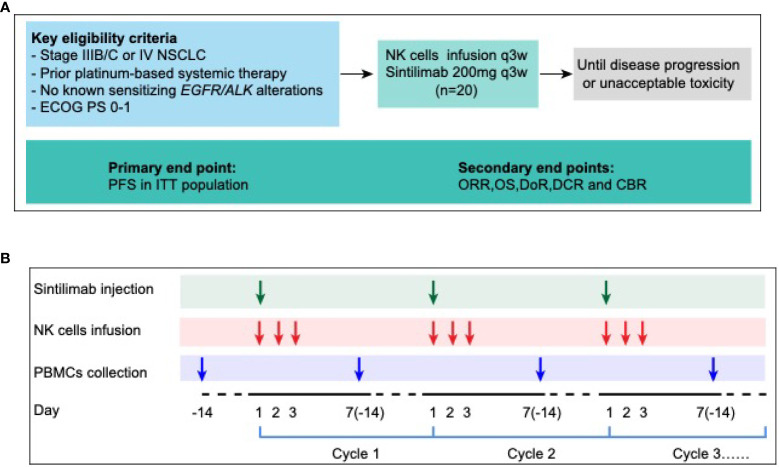
Ensemble diagram. **(A)** Study design. **(B)** Autologous PBMCs were collected by apheresis on D-14, then NK cells were isolated and cultured. 3×10^9^ NK cells were divided into 3 days infusion during D1-D3, 200mg PD-1 antibody (sintilimab) was given on D1, 1 hour after NK cells infusion. NSCLC, non-small-cell lung cancer; ECOG PS, Eastern Cooperative Oncology Group performance status; PFS, progression-free survival; ITT, intention to treatment; ORR, objective response rate; OS, overall survival; DoR, duration of response; DCR, disease control rate; CBR, clinical benefit rate; NK cells, Natural Killer cells; PBMC, peripheral blood mononuclear cells.

This pilot study evaluated the efficacy and safety of anti-PD-1 antibody combined with autologous NK cells in treatment of patients with locally advanced or metastatic NSCLC previously treated with a platinum-containing regimen (**Figure 5A**). Patients received sintilimab 200mg and 3×10^9^ NK cells every 3 weeks until progressive disease, unacceptable toxicity, or withdrawal of consent (14, 17) (**Figure 5B**). The study protocol was approved by institutional review board of first hospital of Jilin University study center, and the study was done in accordance with standards of Good Clinical Practice and the Declaration of Helsinki. All patients provided written informed consent before enrollment. (ClinicalTrials.gov identifier: NCT03958097)

### Sintilimab and expansion of NK cells

Sintilimab (Tyvyt) is a monoclonal antibody against programmed cell death protein 1 (PD-1). It could block the interaction between PD-1 and its ligands and help the anti-tumor effect of T-cells to recover. Sintilimab is developed by Innovent Biologics and Eli Lilly and Company (18). NK cells were expanded by our previously described method (19). Briefly, blood samples were centrifuged at 1800×g for 10 min, and plasma was transferred to new tubes. Peripheral blood mononuclear cells (PBMCs) were isolated by density gradient centrifugation using Ficoll (Axis-Shield PoC AS, Oslo, Norway) at 800×g for 30 min. PBMCs were resuspended in Aly505 medium (Cell Science & Technology Institute Inc., Yamagata, Japan) medium with 5% auto-plasma, 600 U/mL IL-2, and 10 ng/mL IL-15 (both from Miltenyi Biotec, Germany) and 1 μg/mL OK432 (T&L Biological Technology, Beijing, China) at a concentration of 1×10^6^ cells/mL in a humidified atmosphere of 5% CO_2_. The cells were cultured in Aly505 medium supplemented with 5% auto-plasma, 500 U/mL IL-2 and 10 ng/mL IL-15 at 37°C for the following days.

### Patients

Eligible patients were adults with pathologically confirmed locally advanced or metastatic NSCLC who had failed the first line of platinum-based chemotherapy and had at least one measurable lesion by Response Evaluation Criteria in Solid Tumors (RECIST), version 1.1. Eligible patients had to be with an Eastern Cooperative Oncology Group performance status of 0 or 1. Patients with EGFR or ALK mutations were excluded. Patients previously received immunotherapies (ICI, CAR-T cell, antitumor vaccine, etc.) or had a history of severe allergic reactions to other monoclonal antibodies were excluded.

### End points and assessments

The primary end point was PFS. Secondary endpoints included ORR, OS, duration of response (DoR), disease control rate (DCR), clinical benefit rate (CBR) and safety. The exploratory predictive biomarkers were TMB, PD-1 and PD-L1 expression on NK cells.

All treated patients were evaluated for safety since enrolled the study and up to 90 days after receiving the last dose of sintilimab and/or NK cells. The adverse events (AEs) defined by Medical Dictionary for Regulatory Activities Preferred Term and System Organ Class (version 10) and graded based on the National Cancer Institute Common Terminology Criteria for Adverse Events (version 4.03).

After the first dose, we conducted tumor assessments every 6 weeks until progressive disease. The assessment should be carried out by the same assessor to ensure inherent consistency. Objective response should be confirmed by repeated assessments ≥4 weeks after the first observation of response.

### Exploration of predictive markers

Before and at the end of NK cell treatment, the peripheral blood of patients was collected, and stained with mouse monoclonal antibodies against human CD3, CD56, PD-1 and PD-L1 antibodies (Abs). Appropriate isotype-matched Abs were used as controls. 17 patient’s samples were available, including 12 patients in DC group and 5 patients in PD group. The expressions of PD-1 and PD-L1 on NK cells are detected by BD FACS Calibur (BD Biosciences, San Jose, CA, USA). Analysis was performed with FlowJo software (Tree Star, Inc., Ashland, OR, USA).

After progression of one platinum-based regimen, re-biopsy tumor specimens were collected for PD-L1 detection. Next Generation Sequencing, Tumor Mutation Burden and Molecular tumor burden index was analyzed at base line and each time point of tumor assessment by collecting peripheral blood.

#### NGS and sequencing data analysis

ctDNA was isolated using the QIAamp Circulating Nucleic Acid Kit (Qiagen). Sequencing libraries were hybridized to custom-designed biotinylated oligonucleotide probes (Roche NimbleGen) targeting 1,021 genes. Sequencing data were analyzed using default parameters. Contra (v2.0.8) was used to detect copy-number variants (CNVs), and NCsv (in-house software version 0.2.3) was used to detect structural variants (SVs). The final candidate variants were all manually verified using Integrative Genomics Viewer (5).

#### TMB evaluation

TMB was calculated as the number of somatic nonsynonymous single-nucleotide variants (SNVs) and small insertions/deletions per Mb in the coding region (with VAF ≥0.03 for tumor tissue, and ≥0.005 for ctDNA, respectively). TMB-high patients were identified with ≥9 mutations/Mb using the top quartile threshold of 2,000 samples from the Geneplus database (5).

#### Molecular tumor burden index analysis

mTBI was calculated using the mean allele fraction of mutations in a mutation cluster with the highest cellular prevalence of ctDNA at each time point sample. mTBI reflects the percentage of ctDNA detected in cfDNA and its changes can reflect the change of tumor burden at the molecular level (16).

### Statistical analysis

The database lock for the current analyses was Nov 1, 2021. The intent-to-treat (ITT) population comprised all patients who received at least one dose of sintilimab and NK cells. Safety data was summarized for the ITT population and included events reported from enrollment to 90 days after the last dose of sintilimab and/or NK cells. PFS and OS were estimated from the time of enrollment using the Kaplan-Meier methodology, with median values and 2-sided 95% CIs calculated using the Brookmeyer and Crowley method.

## Data availability statement

The original contributions presented in the study are included in the article/**Supplementary Material**, further inquiries can be directed to the corresponding author/s.

## Ethics statement

The studies involving human participants were reviewed and approved by institutional review board of First Hospital of Jilin University study center. The patients/participants provided their written informed consent to participate in this study.

## Author contributions

JC and WL substantially contributed to the conception, design, or planning of the study. LJ, NC, and CN substantially contributed to the cell collection and production. LJ, NC, XC, ZLL, KM, NW, LY, YZ, WS, JL, CC, XFC, XW, YX, GC, ZGL, and JC substantially contributed to acquisition of data. JC, LJ, NC, and RC substantially contributed to analysis of the data. JC, WL, LJ, NC, and RC substantially contributed to interpretation of the results. JC, LJ, and NC substantially contributed to drafting the manuscript. JC, WL, LJ, and NC substantially contributed to critically reviewing or revising the manuscript for important intellectual content. All authors contributed to manuscript revision and approved the submitted version.

## References

[1] Siegel RL Miller KD Fuchs HE Jemal A. Cancer statistics, 2021. CA Cancer J Clin (2021) 71(1):7–33. doi: 10.3322/caac.21654 33433946

[2] Huang MY Jiang XM Wang BL Sun Y Lu JJ. Combination therapy with PD-1/PD-L1 blockade in non-small cell lung cancer: strategies and mechanisms. Pharmacol Ther (2021) 219:107694. doi: 10.1016/j.pharmthera.2020.107694 32980443

[3] Hsu J Hodgins JJ Marathe M Nicolai CJ Bourgeois-Daigneault MC Trevino TN. Contribution of NK cells to immunotherapy mediated by PD-1/PD-L1 blockade. J Clin Invest (2018) 128(10):4654–68. doi: 10.1172/JCI99317 PMC6159991 30198904

[4] Souza-Fonseca-Guimaraes F Cursons J Huntington ND. The emergence of natural killer cells as a major target in cancer immunotherapy. Trends Immunol (2019) 40(2):142–58. doi: 10.1016/j.it.2018.12.003 30639050

[5] Ai X Cui J Zhang J Chen R Lin W Xie C. Clonal architecture of EGFR mutation predicts the efficacy of EGFR-tyrosine kinase inhibitors in advanced NSCLC: A prospective multicenter study (NCT03059641). Clin Cancer Res (2021) 27(3):704–12. doi: 10.1158/1078-0432.CCR-20-3063 33188140

[6] Wu YL Lu S Cheng Y Zhou C Wang J Mok T. Nivolumab versus docetaxel in a predominantly Chinese patient population with previously treated advanced NSCLC: CheckMate 078 randomized phase III clinical trial. J Thorac Oncol (2019) 14(5):867–75. doi: 10.1016/j.jtho.2019.01.006 30659987

[7] Zhou C Huang D Yu X Liu Y Fan Y Shu Y. Abstract CT039: Results from RATIONALE 303: A global phase 3 study of tislelizumab (TIS) vs docetaxel (TAX) as second- or third-line therapy for patients with locally advanced or metastatic NSCLC. Cancer Res (2021) 81(13 Supplement):CT039–CT. doi: 10.1158/1538-7445.Am2021-ct039

[8] Zhang F Huang D Li T Zhang S Wang J Zhang Y. Anti-PD-1 therapy plus chemotherapy and/or bevacizumab as second line or later treatment for patients with advanced non-small cell lung cancer. J Cancer (2020) 11(3):741–9. doi: 10.7150/jca.37966 PMC6959040 31942197

[9] Zhai X Jing X Li J Tian Y Xu S Wang M. Clinical outcomes for PD-1 inhibitor plus chemotherapy as second-line or later therapy compared to PD-1/PD-L1 inhibitor alone in advanced non-small-cell lung cancer. Front Oncol (2020) 10:556275. doi: 10.3389/fonc.2020.556275 33102221 PMC7554577

[10] Morvan MG Lanier LL. NK cells and cancer: you can teach innate cells new tricks. Nat Rev Cancer (2016) 16(1):7–19. doi: 10.1038/nrc.2015.5 26694935

[11] Barry KC Hsu J Broz ML Cueto FJ Binnewies M Combes AJ. A natural killer-dendritic cell axis defines checkpoint therapy-responsive tumor microenvironments. Nat Med (2018) 24(8):1178–91. doi: 10.1038/s41591-018-0085-8 PMC6475503 29942093

[12] Lin M Luo H Liang S Chen J Liu A Niu L. Pembrolizumab plus allogeneic NK cells in advanced non-small cell lung cancer patients. J Clin Invest (2020) 130(5):2560–9. doi: 10.1172/JCI132712 PMC7190908 32027620

[13] Dong W Wu X Ma S Wang Y Nalin AP Zhu Z. The mechanism of anti-PD-L1 antibody efficacy against PD-L1-Negative tumors identifies NK cells expressing PD-L1 as a cytolytic effector. Cancer Discovery (2019) 9(10):1422–37. doi: 10.1158/2159-8290.CD-18-1259 PMC7253691 31340937

[14] Zhou C Wu L Fan Y Wang Z Liu L Chen G. Sintilimab plus platinum and gemcitabine as first-line treatment for advanced or metastatic squamous NSCLC: Results from a randomized, double-blind, phase 3 trial (ORIENT-12). J Thorac Oncol (2021) 16(9):1501–11. doi: 10.1016/j.jtho.2021.04.011 34048947

[15] Sha D Jin Z Budczies J Kluck K Stenzinger A Sinicrope FA. Tumor mutational burden as a predictive biomarker in solid tumors. Cancer Discovery (2020) 10(12):1808–25. doi: 10.1158/2159-8290.CD-20-0522 PMC7710563 33139244

[16] Yi Z Ma F Rong G Liu B Guan Y Li J. The molecular tumor burden index as a response evaluation criterion in breast cancer. Signal Transduct Target Ther (2021) 6(1):251. doi: 10.1038/s41392-021-00662-9 34230452 PMC8260637

[17] Li L Li W Wang C Yan X Wang Y Niu C. Adoptive transfer of natural killer cells in combination with chemotherapy improves outcomes of patients with locally advanced colon carcinoma. Cytotherapy (2018) 20(1):134–48. doi: 10.1016/j.jcyt.2017.09.009 29056549

[18] Zhang L Mai W Jiang W Geng Q. Sintilimab: A promising anti-tumor PD-1 antibody. Front Oncol (2020) 10:594558. doi: 10.3389/fonc.2020.594558 33324564 PMC7726413

[19] Niu C Jin H Li M Xu J Xu D Hu J. *In vitro* analysis of the proliferative capacity and cytotoxic effects of ex vivo induced natural killer cells, cytokine-induced killer cells, and gamma-delta T cells. BMC Immunol (2015) 16:61. doi: 10.1186/s12865-015-0124-x 26458364 PMC4601131

