# Supplementary material for: Sintilimab plus autologous NK cells as second-line treatment for advanced non-small-cell lung cancer previous treated with platinum-containing chemotherapy
Source: Front Immunol. 2022 Dec 8;13:1074906. doi: 10.3389/fimmu.2022.1074906 (PMC9773193; doi:10.3389/fimmu.2022.1074906)
Supplement: Supplementary file 1 [file DataSheet_1.docx]

**Table S1.**

| Subgroup | N, % | ORR | Median PFS, mo  (95% CI) | HR  (95% CI) | P value |
| --- | --- | --- | --- | --- | --- |
| Age, years | | | | | |
| ≥ 65 | 5, 25% | 25.0% | NA (3.57 to NA) | 0.545  (0.12 to 2.49) | 0.434 |
| < 65 | 15, 75% | 46.7% | 11.37 (2.9 to NA) |  |  |
| Smoking status | | | | | |
| Smoker | 10, 50% | 30.0% | 15.78 (5.1 to NA) | 0.707  (0.23 to 2.21) | 0.552 |
| Never | 10, 50% | 60.0% | 3.23 (2.0 to NA) |  |  |
| Histology | | | | | |
| Adenocarcinoma | 8, 40% | 12.5% | 2.02 (1.97 to NA) | 3.39  (1.06 to 10.8) | 0.039^*^ |
| Squamous | 12, 60% | 66.7% | 19.67(11.37 to NA) |  |  |
| TMB | | | | | |
| < 9 mut/Mb | 14, 70% | 35.7% | 8.50 (3.57 to NA) | 1.16  (0.31 to 4.33) | 0.821 |
| ≥ 9 mut/Mb | 6, 30% | 66.7% | 11.37 (1.97 to NA) |  |  |
|  |  |  |  |  |  |

**Table S1.** ORR and PFS by Baseline Characteristic Subgroups

Abbreviation: NA, not applicable. ^*^P<0.05

**Fig. S1.**

**Fig. S1.** Overall survival in intention to treat population.

**Table S2.**

| Event, No. (%) | All Patients (N = 20) | | | |
| --- | --- | --- | --- | --- |
|  | Any Grade | Grade 1 | Grade 2 | Grade 3 |
| Any event | 19 (95) | 11(55) | 6(30) | 2(10) |
| Any event leading to discontinuation | 1(5) | 0 (0) | 1(5) | 0 (0) |
| Most frequent events (≥10%) |  |  |  |  |
| Hypoalbuminemia | 9 (45) | 8(40) | 1(5) | 0 (0) |
| Hypothyroidism | 5 (25) | 4(20) | 1(5) | 0 (0) |
| Anemia | 4 (20) | 2(10) | 2(10) | 0 (0) |
| Hyperglycemia | 4 (20) | 3(15) | 1(5) | 0 (0) |
| Hyponatremia | 4 (20) | 4(20) | 0(0) | 0 (0) |
| Hyperuricemia | 3 (15) | 3(15) | 0(0) | 0 (0) |
| Hyperthyroidism | 3 (15) | 3(15) | 0(0) | 0 (0) |
| Alkaline phosphatase increased | 3 (15) | 3(15) | 0(0) | 0 (0) |
| CPK increased | 2 (10) | 0(0) | 1 (5) | 1 (5) |
| Hypocalcemia | 2 (10) | 1(5) | 1(5) | 0 (0) |
| Platelet count decreased | 2 (10) | 2 (10) | 0 (0) | 0 (0) |
| AST increased | 2 (10) | 2 (10) | 0 (0) | 0 (0) |
| Proteinuria | 2 (10) | 2 (10) | 0 (0) | 0 (0) |
| Cough | 2 (10) | 2 (10) | 0 (0) | 0 (0) |
| Hypertriglyceridemia | 1 (5) | 0 (0) | 0 (0) | 1 (5) |
| Neutrophil count decreased | 1 (5) | 0 (0) | 0 (0) | 1 (5) |
| WBC decreased | 1 (5) | 0 (0) | 1 (5) | 0 (0) |
| Rash | 1 (5) | 0 (0) | 1 (5) | 0 (0) |
| Interstitial lung disease | 1 (5) | 0 (0) | 1 (5) | 0 (0) |
| Alanine aminotransferase increased | 1 (5) | 1 (5) | 0 (0) | 0 (0) |
| Thyroiditis | 1 (5) | 1 (5) | 0 (0) | 0 (0) |
| Increased creatinine | 1 (5) | 1 (5) | 0 (0) | 0 (0) |
| Hypercholesterolemia | 1 (5) | 1 (5) | 0 (0) | 0 (0) |

**Table S2.** Frequency of Adverse Events

**Fig. S2.**

**Fig. S2.** Baseline TMB level. DC group: Disease control group, with best response of CR, PR and SD. PD group: Progressive disease group, with best response of PD.

**Fig. S3.**

1. **B.**

**
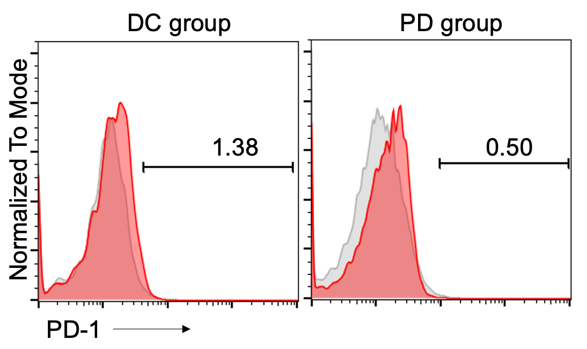

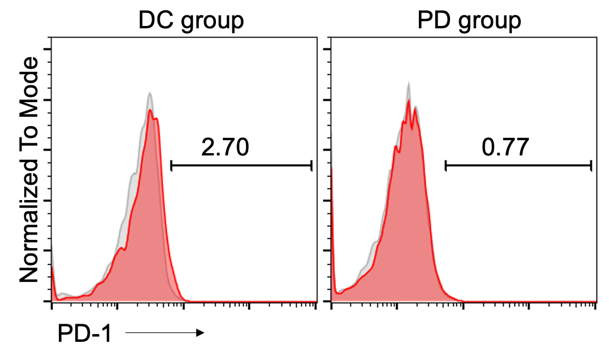
**

1. **D.**

**Fig. S3.** PD-1 expression of NK cells. (A) Representative flow cytometry analysis of PD-L1 expression at baseline. (B) Representative flow cytometry analysis of PD-L1 expression after treatment. (C) PD-1 of NK cells had no significant difference between DCR group and PD group both at baseline(A) and after treatment(B).

**Fig. S4.**

**A.**

mm

**B.**


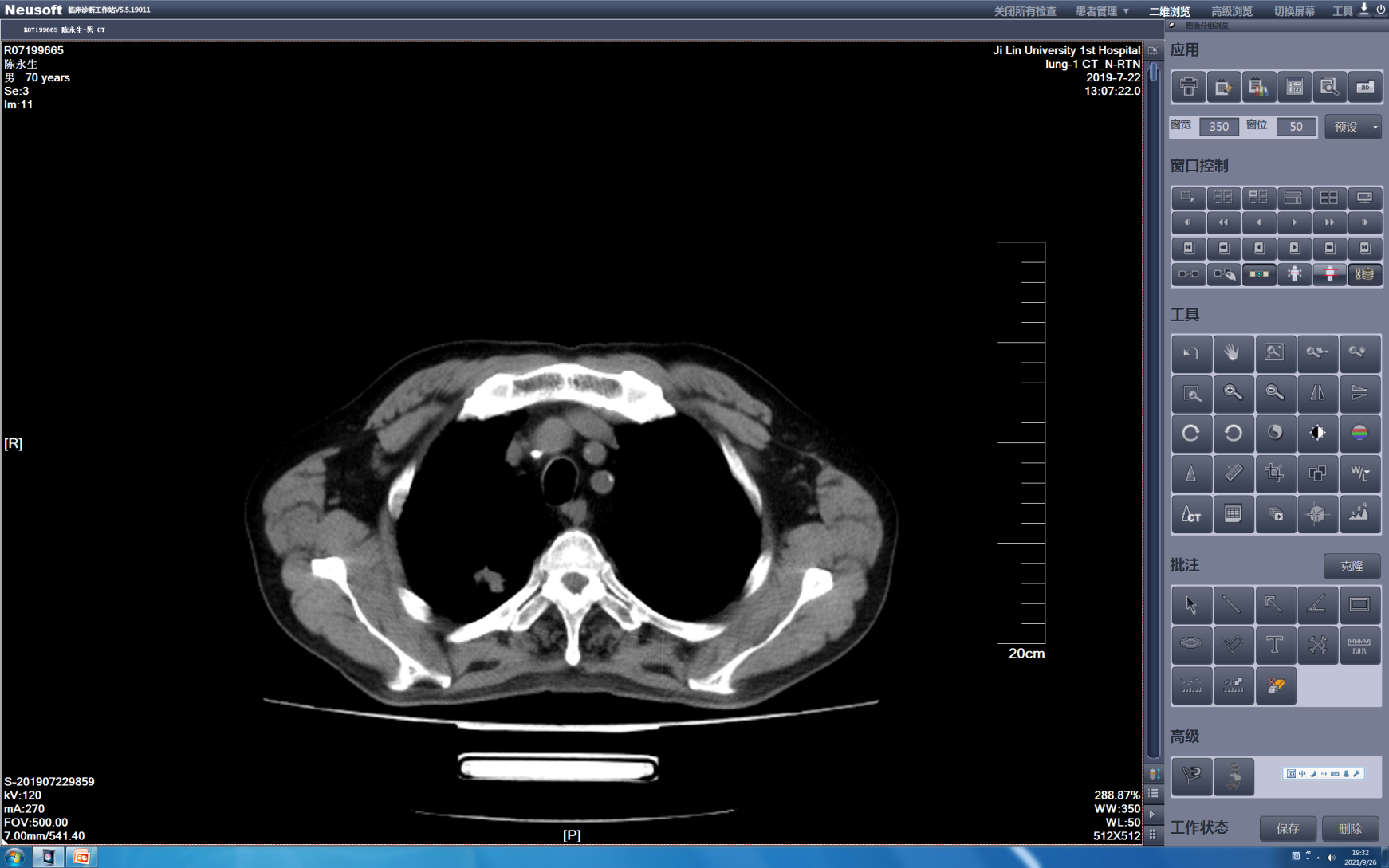

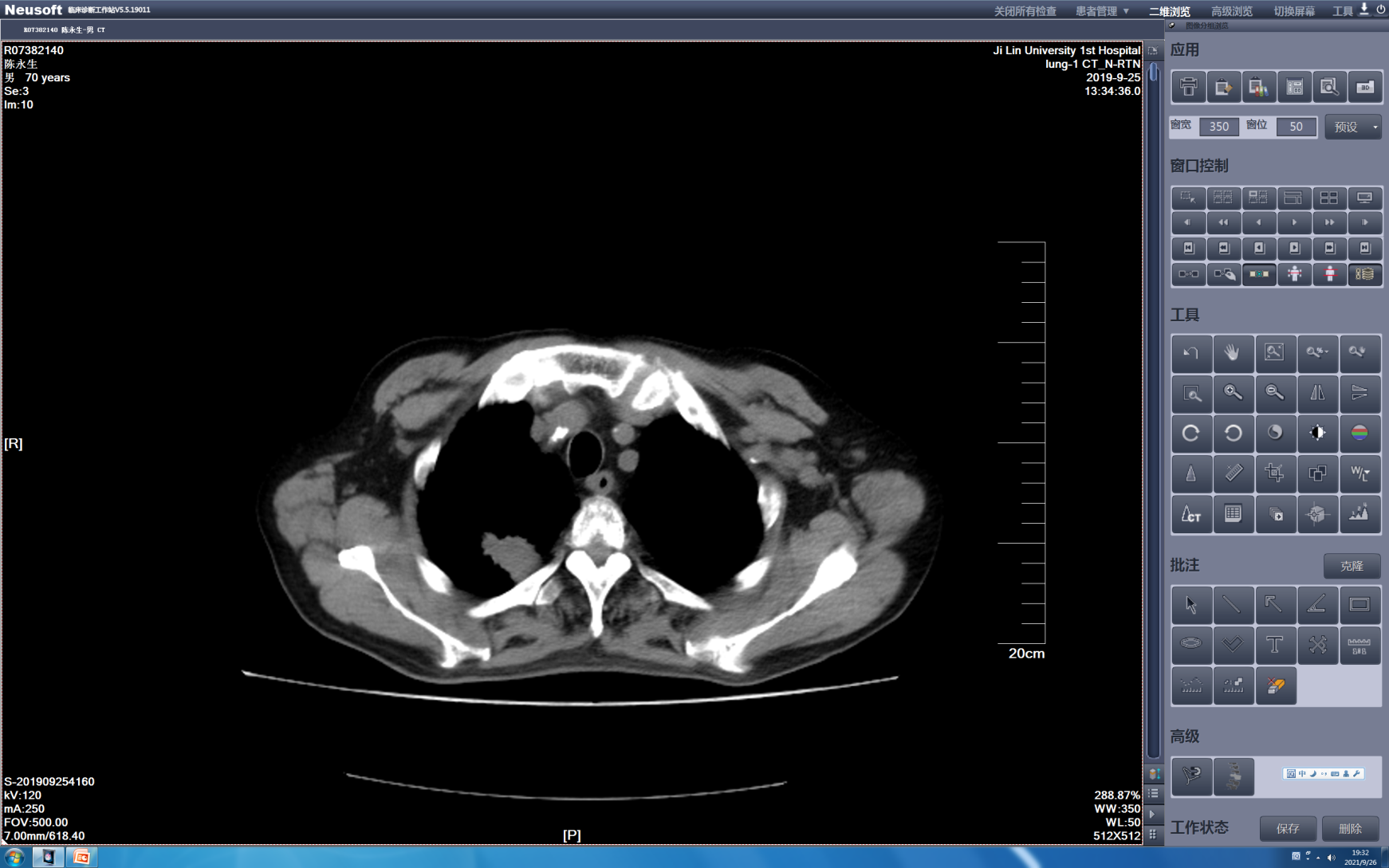


baseline

C2

**Fig. S4.** Patient 01, who got disease progression just after 2 cycles treatment. mTBI(A) increased accompanied with disease progression(B).

**Fig. S5.**

**A.**

mm

**B.**


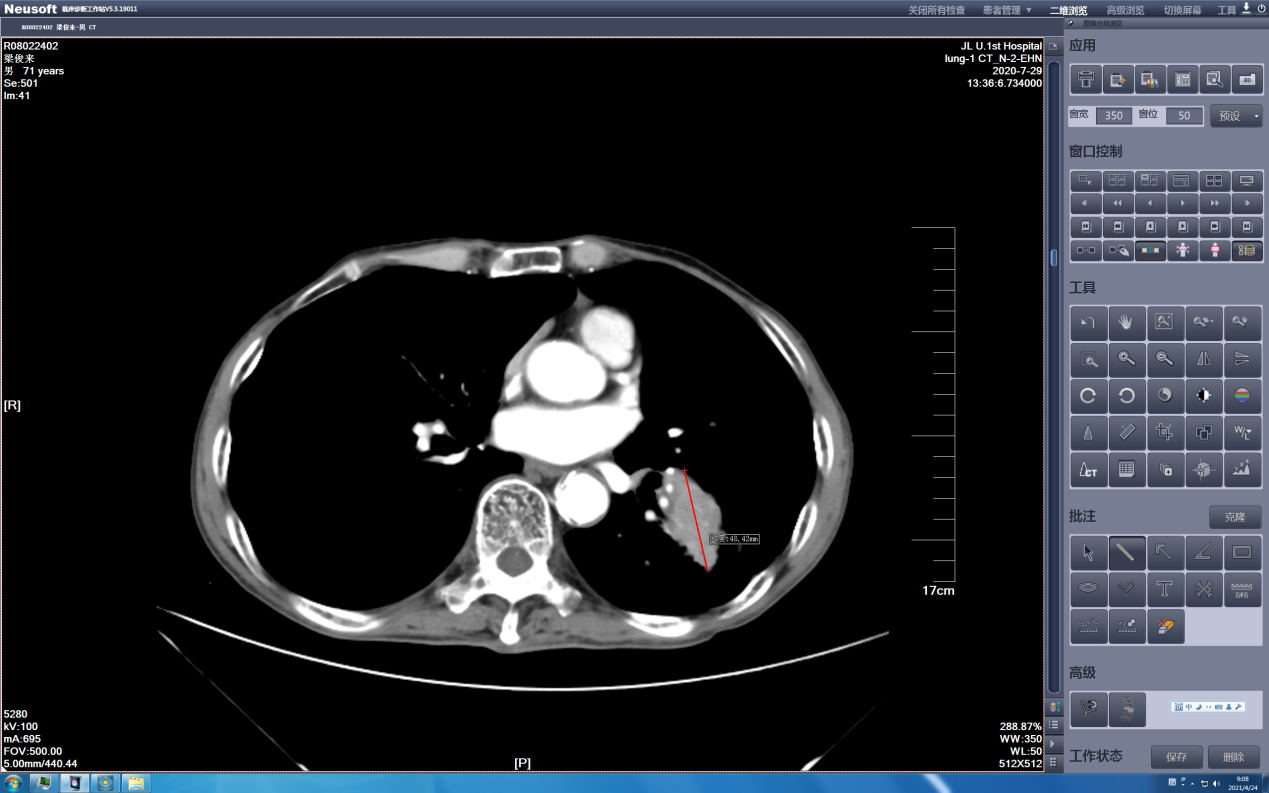

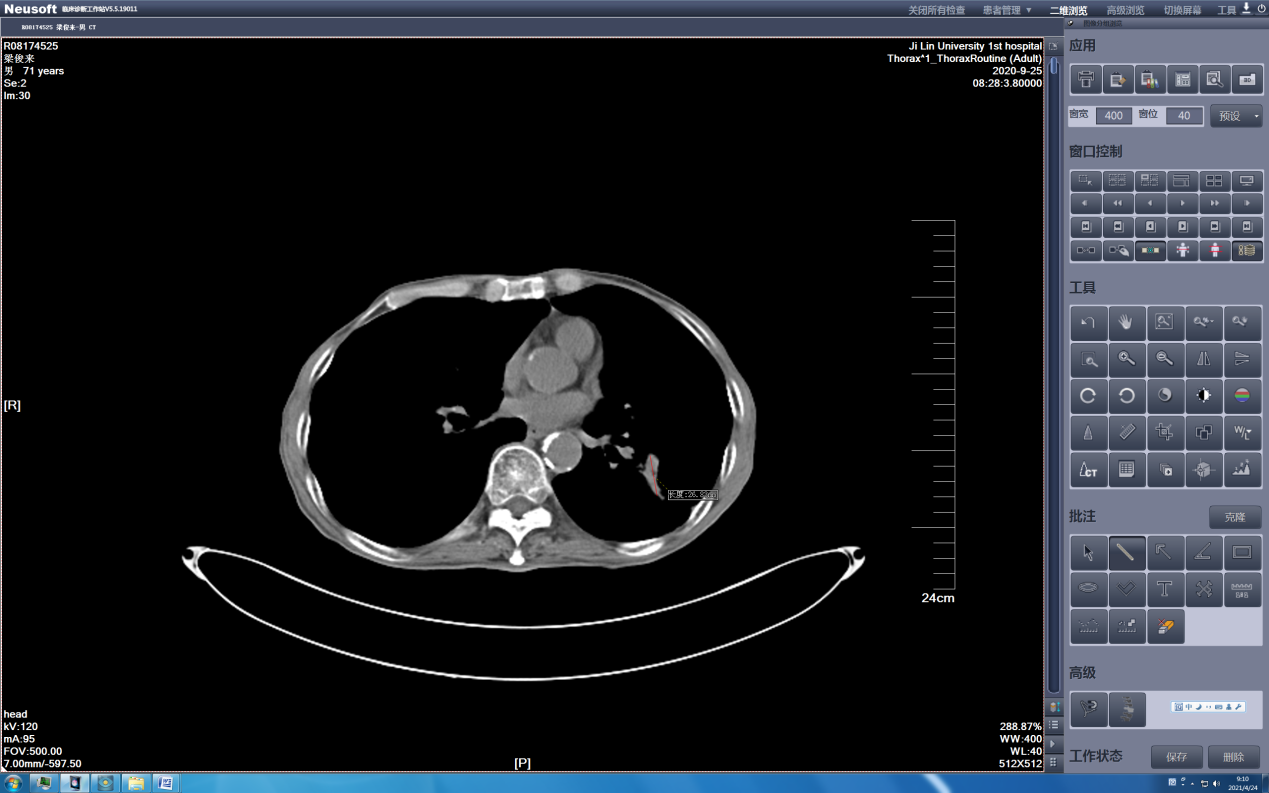

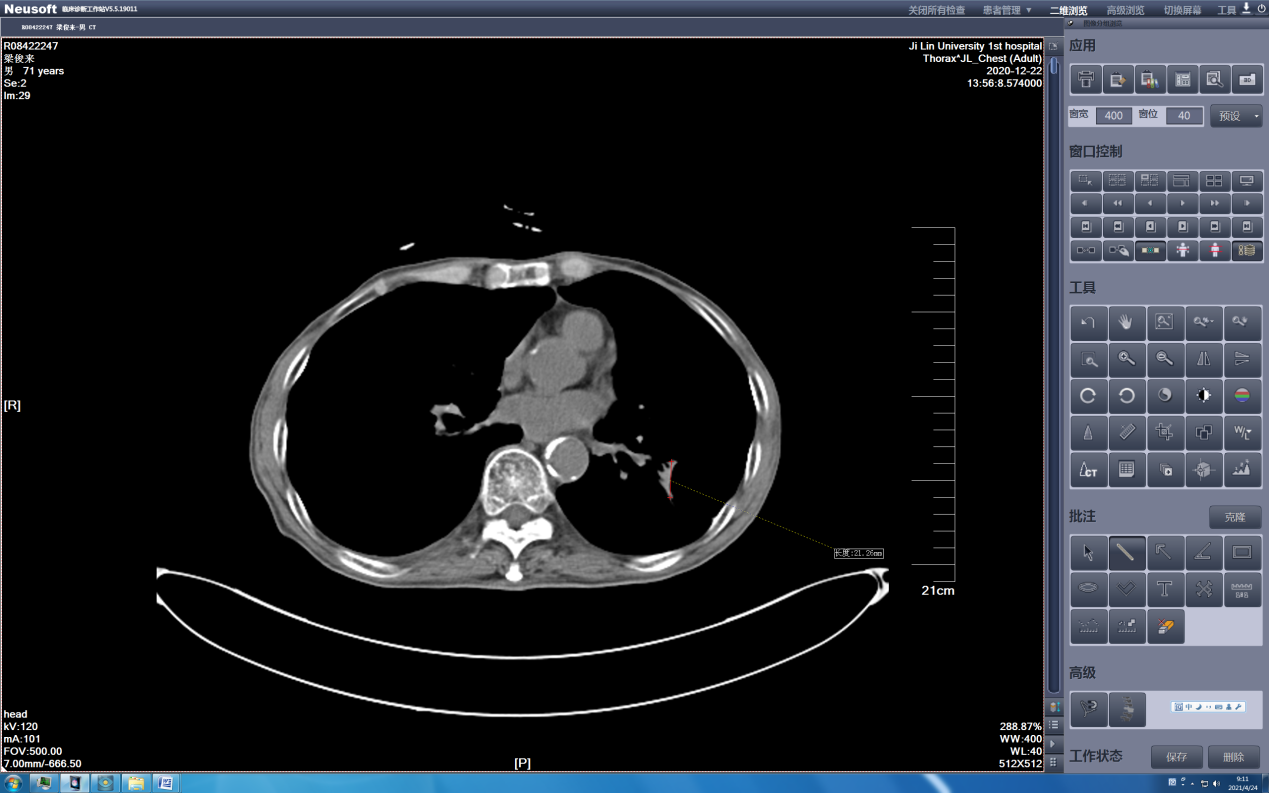


C6

C2

baseline

**Fig. S5.** Patient 017, whose best response status was PR. mTBI(A) decreased when tumor size reduced(B).

**Fig. S6.**

**A.**

mm

**B.**


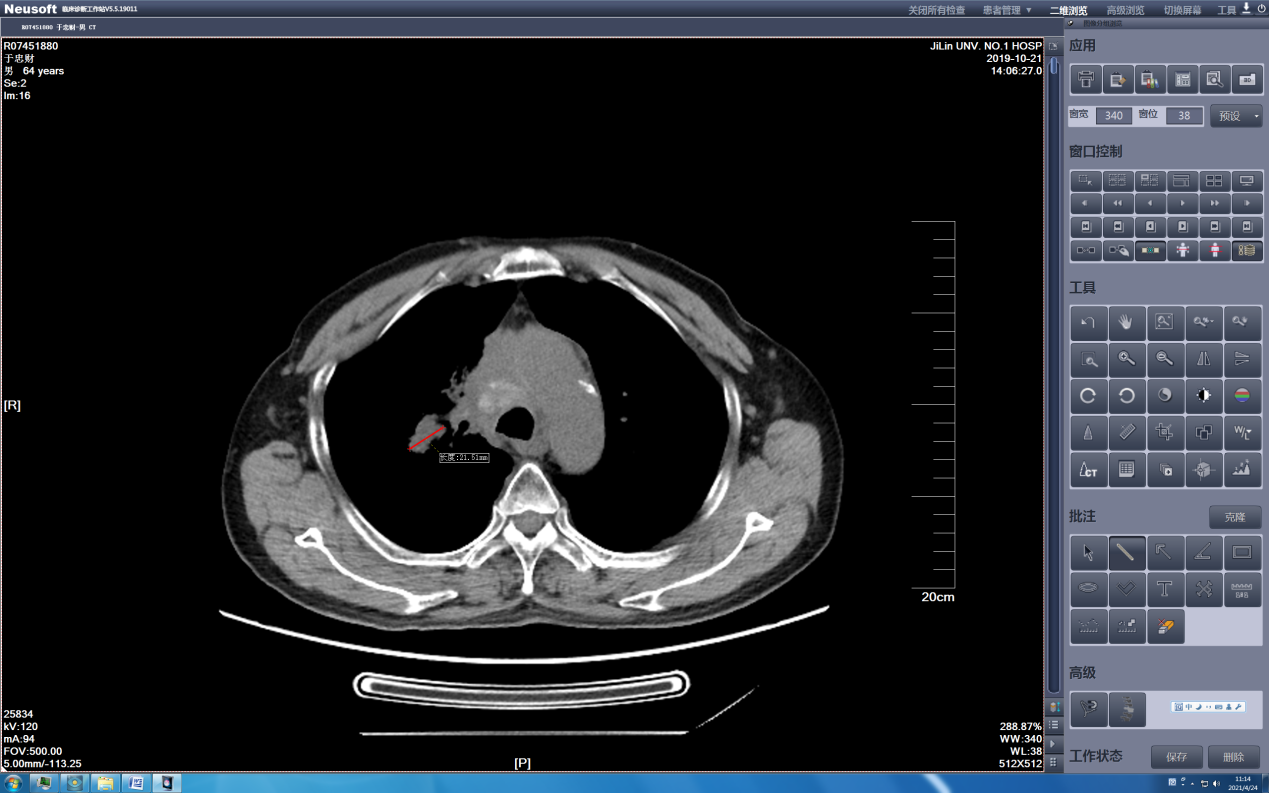

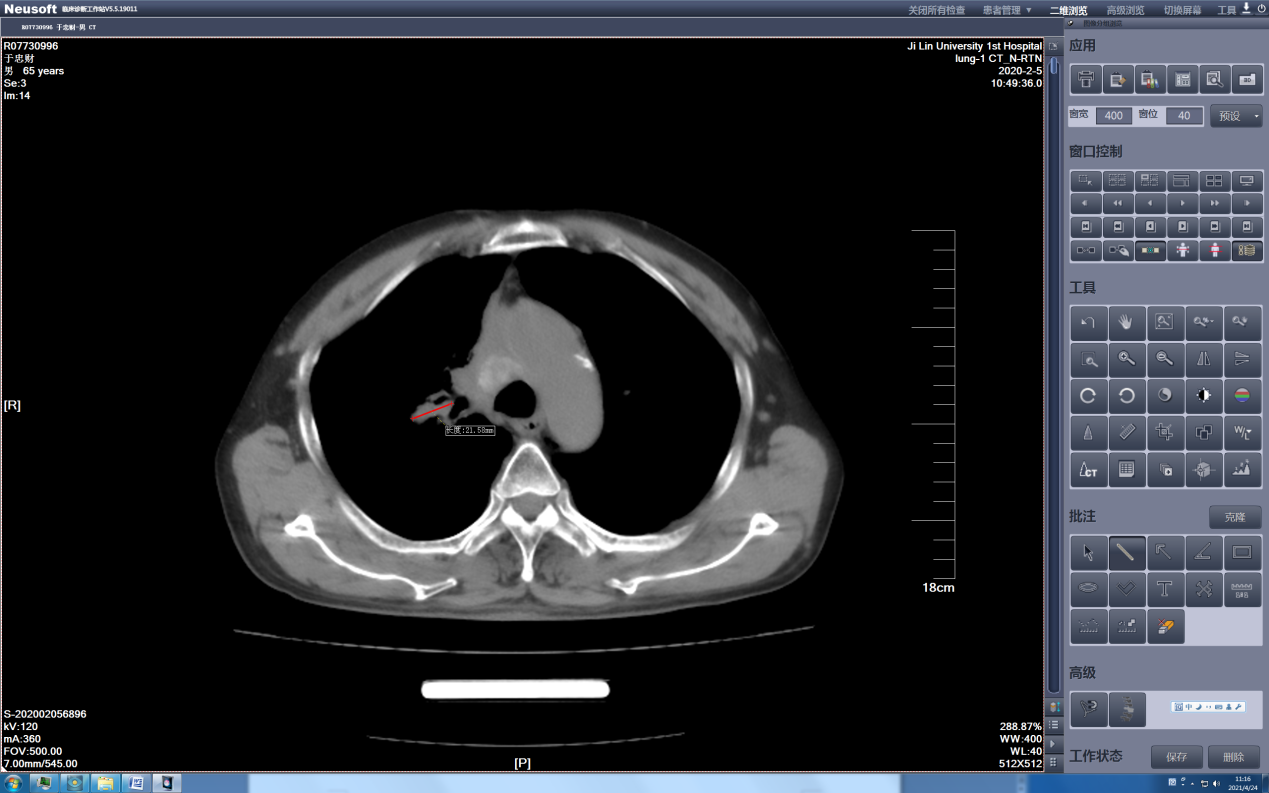

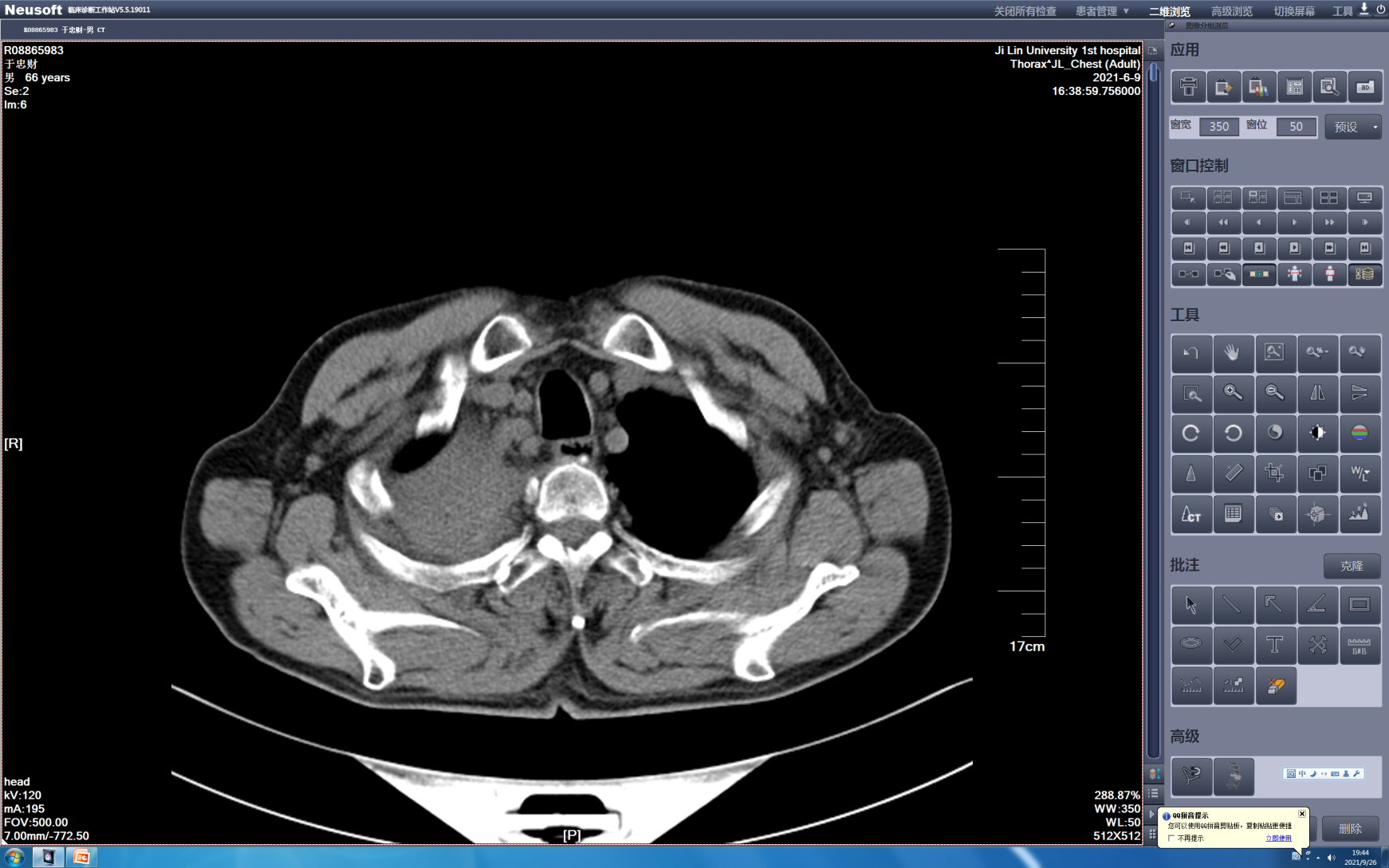


C24

C8

baseline

**Fig. S6.** Patient 008, whose best response status was SD. Target lesion remained stable, but mTBI increased continually(A). After 20 cycles treatment, right malignant pleural effusion emerged(B).
